# Additive negative effects of anthropogenic sedimentation and warming on the survival of coral recruits

**DOI:** 10.1038/s41598-017-12607-w

**Published:** 2017-09-28

**Authors:** Francesca Fourney, Joana Figueiredo

**Affiliations:** Nova Southeastern University, Halmos College of Natural Sciences and Oceanography, Dania Beach, FL 33004 USA

## Abstract

Corals worldwide are facing population declines due to global climate change and local anthropogenic impacts. Global climate change effects are hard to tackle but recent studies show that some coral species can better handle climate change stress when provided with additional energy resources. The local stressor that most undermines energy acquisition is sedimentation because it impedes coral heterotrophic feeding and their ability to photosynthesize. To investigate if reducing local sedimentation will enable corals to better endure ocean warming, we quantitatively assessed the combined effects of increased temperature and sedimentation (concentration and turbidity) on the survival of coral recruits of the species, *Porites astreoides*. We used sediment from a reef and a boat basin to mimic natural sediment (coarse) and anthropogenic (fine) sediment (common in dredging), respectively. Natural sediment did not negatively impact coral survival, but anthropogenic sediment did. We found that the capacity of coral recruits to survive under warmer temperatures is less compromised when anthropogenic sedimentation is maintained at the lowest level (30 mg.cm^−2^). Our study suggests that a reduction of US-EPA allowable turbidity from 29 Nephelometric Turbidity Units (NTU) above background to less than 7 NTU near coral reefs would facilitate coral recruit survival under current and higher temperatures.

## Introduction

Coral reefs are one of the most important and biologically diverse ecosystems on Earth. These habitats provide several essential ecosystem services and have significant economic value. Scleractinian corals construct the foundation of the reef by excreting calcium carbonate skeletons, and provide habitat, shelter, nursery areas, and/or food for thousands of plant and animal species^1^. Coral reefs are essential in sustaining the livelihood of many coastal communities through the supply of seafood (fisheries), raw materials for medicines, and income through tourism^2^. Reefs also provide important ecosystem services such as physical stabilization of the shoreline and control of the carbon dioxide-calcium budget^2^.

Corals face multiple natural stressors, but the worldwide population declines registered in the past decades are mostly due to anthropogenic impacts^3–8^. Natural stressors include predation, intra and interspecific competition for space, and severe storms. Corals co-evolved with predators and competitors, thus their coexistence should be ecologically stable. Likewise, severe storms physically destroy reefs, however corals evolved in the storm-prone tropics, and thus, in the absence of anthropogenic stressors, corals should be able to recover from these disturbances through natural processes^9^. Recovery after disturbances is dependent on the growth of the surviving coral population through asexual reproduction and/or recruitment through sexual reproduction. Successful coral recruitment requires larval availability, successful settlement, and post-settlement survival and growth^10^. The slow or lack of recovery after disturbances observed in some coral reefs in the past few decades^11^ indicates that these natural recovery processes have been undermined by anthropogenic impacts, such as nutrient^8,12^ and sediment^13,14^ runoff from sewage and land development, and the loss of herbivores due to overfishing^15^ or die off from disease (e.g. sea urchin, *Diadema antillarum*)^16^. After a certain threshold, the combination of anthropogenic and natural stressors causes major phase shifts from coral-dominated reefs to less desirable states, such as macroalgae or sponge dominated reef ecosystems^7,16,17^.

Dredging is particularly stressful for corals as it increases turbidity and sedimentation. Although sedimentation occurs naturally through wind-driven waves and tidal currents, dredging can increase the duration, severity, and frequency of the sedimentation^18,19^, with detrimental consequences for coral reefs^14,20,21^. Fine sediment (<125 μm) have the most harmful effects on corals^14,21^. Natural sediment found on the reef usually has larger grain sizes compared to anthropogenic sediment from construction activities. When turbidity is increased due to the presence of fine particles in the water column, light availability is significantly reduced^14^. Higher levels of turbidity negatively impact corals. Corals obtain much of their energy from endosymbiotic algae *Symbiodinium*. Under low light conditions, *Symbiodinium* have lower capacity to photosynthesize and therefore provide less energy to the coral host^3,20,22^. Landfills, beach re-nourishment, and coastal construction can also cause burial and physical disturbance of corals^14,18,21^. Sedimentation can directly smother corals and block polyps, therefore reducing active feeding mechanisms and depleting energy reserves^14^. In these situations, energy typically used for growth and reproduction is allocated towards clearing sediments that have settled on top of the coral subsequently blocking polyp-feeding structures^14^, lowering calcification rates^14,23^ and reproductive output^3,14,24^.

Global climate change has introduced additional stressors to coral reefs. Human activities have increased the atmospheric concentrations of greenhouse gasses, such as carbon dioxide (CO_2_), methane (CH_4_), and nitrous oxide (N_2_O), causing global temperatures to rise^25^. Climate change models predict that tropical sea surface temperatures will rise up to 3 °C this century^25^. Increased seawater temperatures lead to coral bleaching^26,27^, i.e. corals lose their endosymbiotic algae, *Symbiodinium*. These symbionts typically provide about 95% of the energy for the coral^7,28^. In their absence, coral tissue growth, fecundity, calcification, and overall survival are severely degraded^27–29^. Recent studies project coral bleaching will become an annual event within the next 25–50 years, potentially hampering reef recovery and thus, causing exponential declines of coral populations and threatening the persistence of entire reef ecosystems worldwide^28^.

To maintain or increase current coral cover, anthropogenic stressors (including climate change) would have to be reduced. Moderation and strict management of CO_2_ emissions and other greenhouse gases would need to be implemented worldwide^30^; however, global consensus has been difficult to achieve. It may be possible that some corals will be able to tolerate small increases in temperature or slightly more acidified waters, if their ability to acquire energy is not hampered. Studies have shown that some species of coral and fish can buffer deleterious effects of ocean acidification and increased temperatures when additional energy resources (feeding) were provided^31,32^.

This study investigates if reducing sedimentation (and turbidity), a local anthropogenic stressor which hampers considerably the ability of corals to acquire energy^20^, can facilitate coral survival under warmer conditions. To fulfil this goal, we assessed the synergistic effect of sedimentation and elevated temperatures on the survival of coral recruits. We used two types of sediment, natural (coarse) and dredging sediment (with a greater proportion of fine particles, hereafter termed anthropogenic) and tested the effect of sediment concentration ranging from naturally occurring in healthy coral reefs to near dredging operations, which lead to differing levels of turbidity. We used temperatures that mimic the summer average temperature in northern section of the Florida Keys (in years without coral bleaching) and elevated temperatures predicted by climate change models. These effects were tested on coral recruits, since this is the most critical stage for coral recovery^33^ and thus should provide better insight into coral persistence through ocean warming. The results of this study inform managers on critical levels of sedimentation for the persistence of corals now and in the future.

## Results

**Figure 1:**
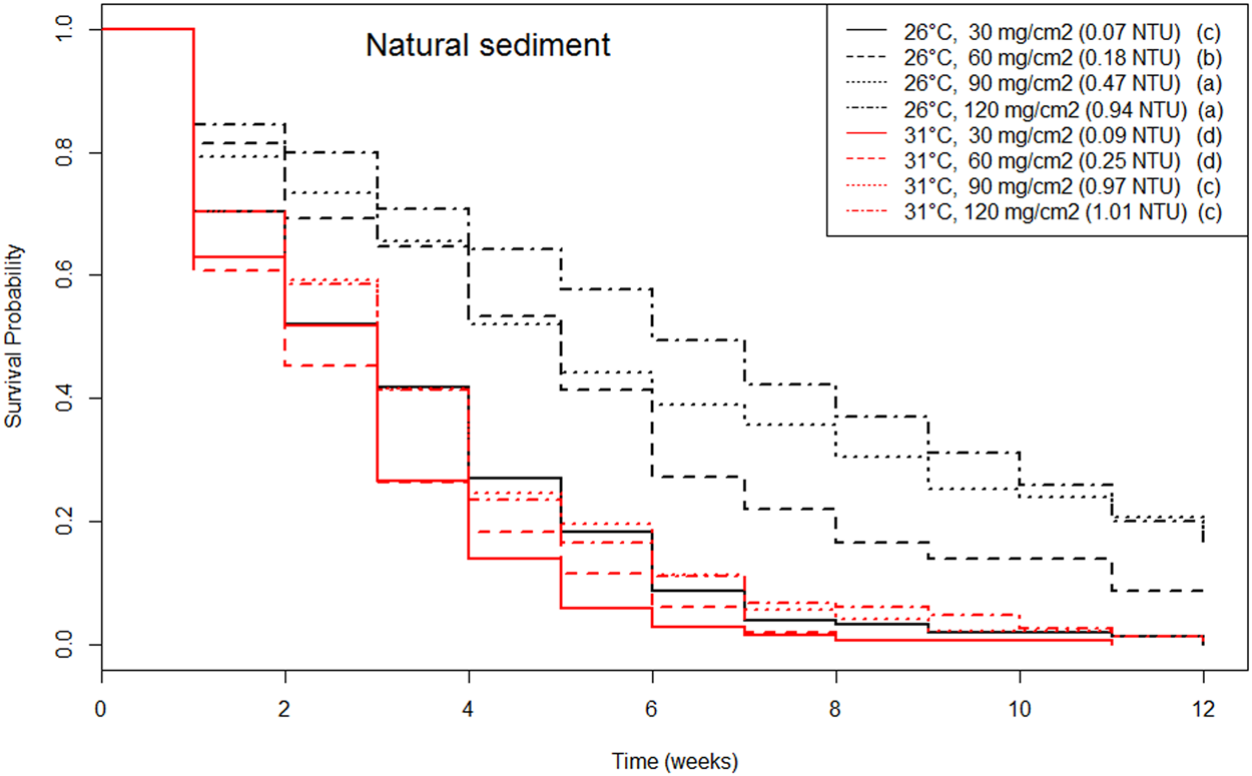
Kaplan-Meier survival curves of recruits at all combinations of temperature and natural sedimentation. Color represents different sediment levels. Smooth lines represent temperature 26 °C and dashed lines represent 30 °C. Letters in the legend indicate homogeneous groups (i.e. treatments with non-significant differences, with group *a* having the highest survival and *d* the lowest).

**Figure 2:**
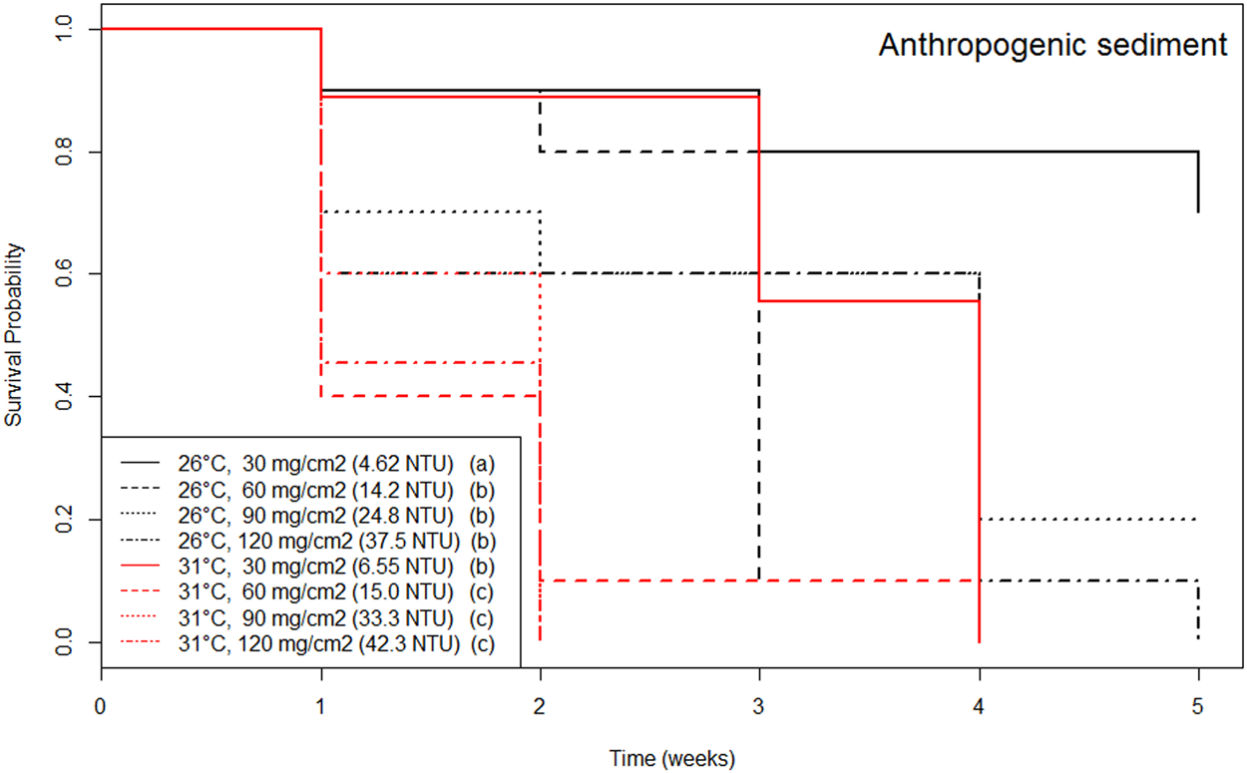
Kaplan-Meier survival curves of recruits at all combinations of temperature and anthropogenic sedimentation. Color represents different sediment level. Smooth lines represent temperature 26 °C and dashed lines represent 30 °C. Letters in the legend indicate homogeneous groups (i.e. treatments with non-significant differences, with group *a* having the highest survival and *d* the lowest).

Coral recruit survival was always significantly lower at increased temperature (p < 2.2 × 10^−16^ and 5.8 × 10^−5^, respectively for natural and anthropogenic sediment). The effect of sedimentation was dependent on sediment type. An increase in natural sediment significantly increased survival of coral recruits (p = 2.2 × 10^−13^, Fig. 1, Table 1), while an increase in anthropogenic sediment significantly reduced coral recruit survival (p = 0.000674, Fig. 2, Table 2).

**Table 1:** Percent survival (±S.E.) over time for all combinations of temperature and deposited sediment concentration using natural sediment.

Time (weeks)	26 °C				30 °C			
	30 mg.cm^−2^	60 mg.cm^−2^	90 mg.cm^−2^	120 mg.cm^−2^	30 mg.cm^−2^	60 mg.cm^−2^	90 mg.cm^−2^	120 mg.cm^−2^
1	70.3 ± 0.4	81.3 ± 0.3	79.2 ± 0.3	84.4 ± 0.3	63.0 ± 0.4	60.8 ± 0.4	70.4 ± 0.4	70.3 ± 0.4
2	52.0 ± 0.4	69.3 ± 0.4	73.4 ± 0.4	79.9 ± 0.3	51.9 ± 0.4	45.3 ± 0.4	59.2 ± 0.4	58.6 ± 0.4
3	41.9 ± 0.4	64.7 ± 0.4	65.5 ± 0.4	70.8 ± 0.4	26.7 ± 0.4	26.4 ± 0.4	41.6 ± 0.4	41.4 ± 0.4
4	27.0 ± 0.4	53.3 ± 0.4	51.9 ± 0.4	64.3 ± 0.4	14.1 ± 0.3	18.2 ± 0.3	24.7 ± 0.4	23.5 ± 0.4
5	18.2 ± 0.3	41.3 ± 0.4	44.2 ± 0.4	57.8 ± 0.4	5.9 ± 0.2	11.5 ± 0.3	19.7 ± 0.3	16.6 ± 0.3
6	8.8 ± 0.2	27.3 ± 0.4	39.0 ± 0.4	49.4 ± 0.4	3.0 ± 0.1	6.1 ± 0.2	11.3 ± 0.3	11.0 ± 0.3
7	4.1 ± 0.2	22.0 ± 0.3	35.7 ± 0.4	42.2 ± 0.4	1.5 ± 0.1	2.0 ± 0.1	5.6 ± 0.2	6.9 ± 0.2
8	3.4 ± 0.2	16.7 ± 0.3	30.5 ± 0.4	37.0 ± 0.4	0.7 ± 0.1	0.0 ± 0.0	4.2 ± 0.2	6.2 ± 0.2
9	2.0 ± 0.1	14.0 ± 0.3	25.3 ± 0.4	31.2 ± 0.4	0.7 ± 0.1	0.0 ± 0.0	2.1 ± 0.1	4.8 ± 0.2
10	2.0 ± 0.1	14.0 ± 0.3	24.0 ± 0.3	26.0 ± 0.4	0.7 ± 0.1	0.0 ± 0.0	2.1 ± 0.1	2.8 ± 0.1
11	1.4 ± 0.1	8.7 ± 0.2	20.8 ± 0.3	20.1 ± 0.3	0.0 ± 0.0	0.0 ± 0.0	1.4 ± 0.1	1.4 ± 0.1
12	0.0 ± 0.0	0.7 ± 0.2	18.2 ± 0.3	16.9 ± 0.3	0.0 ± 0.0	0.0 ± 0.0	1.4 ± 0.1	0.7 ± 0.1

**Table 2:** Percent survival (±S.E.) over time for all combinations of temperature and deposited sediment concentration using anthropogenic sediment.

Time (weeks)	26 °C				30 °C			
	30 mg.cm^−2^	60 mg.cm^−2^	90 mg.cm^−2^	120 mg.cm^−2^	30 mg.cm^−2^	60 mg.cm^−2^	90 mg.cm^−2^	120 mg.cm^−2^
1	90.0 ± 0.9	90.0 ± 0.9	70.0 ± 1.5	60.0 ± 1.5	88.9 ± 1.1	40.0 ± 1.5	60.0 ± 1.6	45.5 ± 1.5
2	90.0 ± 0.9	80.0 ± 1.3	60.0 ± 1.6	60.0 ± 1.5	88.9 ± 1.1	10.0 ± 0.9	0.0 ± 0.0	0.0 ± 0.0
3	80.0 ± 1.3	10.0 ± 0.9	60.0 ± 1.6	60.0 ± 1.5	55.6 ± 1.7	10.0 ± 0.9	0.0 ± 0.0	0.0 ± 0.0
4	80.0 ± 1.3	0.0 ± 0.0	20.0 ± 1.3	10.0 ± 0.9	0.0 ± 0.0	0.0 ± 0.0	0.0 ± 0.0	0.0 ± 0.0
5	70.0 ± 1.4	0.0 ± 0.0	20.0 ± 1.3	0.0 ± 0.0	0.0 ± 0.0	0.0 ± 0.0	0.0 ± 0.0	0.0 ± 0.0

With natural (coarse) sediment, the *Porites astreoides* recruits survived better at ambient temperatures and higher natural (coarse) sedimentation. Increasing temperature to 30 °C reduced survival to levels similar to the ones obtained at ambient temperature and lower natural (coarse) sedimentation (Fig. 1).

Temperature and anthropogenic (fine) sedimentation additively affected the survival of *P. astreoides* recruits. Increasing both temperature and anthropogenic sedimentation led to higher mortality than increasing just one of the stressors, e.g., recruits reared at higher temperatures and anthropogenic sedimentation (30 °C and 60 mg.cm^−2^) displayed significantly higher mortality than recruits reared at lower temperature and similar anthropogenic sedimentation (26 °C and 60 mg.cm^−2^) or recruits reared at similar temperature and lower anthropogenic sedimentation (30 °C and 30 mg.cm^−2^) (Fig. 2).

The negative impacts of higher temperatures can be ameliorated when anthropogenic sedimentation is kept at lower concentrations. Survival of coral recruits is highest at 26 °C and lowest anthropogenic sediment concentration (30 mg.cm^−2^). At higher temperature (30 °C) and lowest anthropogenic sediment concentration (30 mg.cm^−2^), survival decreases to levels not significantly different from the ones obtained at ambient temperatures (26 °C) and higher anthropogenic sediment concentrations (60, 90 or 120 mg.cm^−2^) (Fig. 2).

## Discussion

The survival of *Porites astreoides* recruits under warm conditions can be ameliorated if anthropogenic sedimentation and turbidity are kept at low levels. Higher temperatures naturally increased mortality of coral recruits. The effect of sediment concentration on coral recruits seemed however dependent on the composition of the sediment. High concentration of natural (coarse) sediment was not detrimental to coral recruits, but in fact benefitted them. The opposite happened with anthropogenic (fine) sediment. Increasing amounts of anthropogenic sediment considerably increased turbidity and increased coral recruit mortality. While both temperature and anthropogenic sediment negatively impacted coral recruits, the exclusion of one of the stressors allowed coral recruits to cope better with the other stressor. Importantly, corals under warm conditions (30 °C) but low anthropogenic sedimentation (30 mg.cm^−2^, 6.55 NTU) survived similarly to coral recruits kept at current spring temperatures (26 °C) and higher anthropogenic sedimentation (≥60 mg.cm^−2^, ≥14.2 NTU).

Natural sediment aided the survival of corals recruits. The grain size of natural/reef sediment is coarse. Large grain sediment settled quickly and thus, turbidity remained very low (≤1 NTU, Table 1), as is typical on undisturbed reefs^23^. Instead of detrimental, the coarse sediment settled on top of the coral recruits (completely covering them at highest sediment concentrations) reduced their mortality, likely due to conferring protection from excessive light. This study used a level of light irradiance that mimicked natural conditions on the reef (~240 μmol photons.m^−2^ s^−2^), which is ideal for adult corals. However, the ideal light irradiance for adult corals is often deleterious for newly settled corals^29^. This is not surprising considering the well-known preference of coral larvae to settle on crevices and the downward face of settlement tiles, where they are shaded^34,35^. The reasons for this remain to be understood, but it is possible that at this early stage of development, corals still have relatively small feeding structures and thus cannot provide enough substrate for *Symbiodinium* to perform photosynthesis, leading either to a depletion of their energy reserves (*Symbiodinium* burden hypothesis) or oxidative stress, similar to the one caused by increased temperatures^29,36^. Natural sediment provides cryptic covering from excessive light due to its coarser grain sizes, while still allowing water to flow around the coral recruit and therefore does not limit the availability of oxygen^14^ nor access to food. Previous experiments testing the effect of sedimentation on coral recruits in a shallow area assumed that the higher survival of coral juveniles placed in tiles facing downward (vs. vertical and upward) was reflective of lower exposure to sedimentation, and overlooked the reduced light exposure as a possible explanation for the higher survival^37,38^. The observation that corals tend to successfully recruit facing downwards in shallow water, vertically at intermediate depths and facing upwards at greater depths in both the Caribbean^39^ and Pacific^38^ is also supportive of the hypothesis that light is deleterious during early development. Therefore, we conclude that, within the range tested, natural/coarse sediment is not deleterious for corals.

In contrast to natural sediment, anthropogenic sediment hindered the survival of coral recruits. Anthropogenic sediment arising from dredging, construction, and development activities is mostly small grained (<180 µm), and often contains silt (<63 µm) and contaminants^14,19^. Small-grained particles become easily suspended in the water column, increasing turbidity and therefore corals’ access to light (Table 1). A decreased access to light is problematic for adult corals because it reduces the capacity of the symbiotic algae S*ymbiodinium* to photosynthesize and thus feed the coral^14,23^. For coral recruits, an increase in turbidity may actually offer protection from excessive light. Regardless, high levels of turbidity are indicative that the type of sediment that is covering the corals is deleterious. Fine sediment (silt) can smother corals, particularly coral recruits, because it damages the coral tissue and reduces the access to oxygen creating anoxic conditions that suffocate them and facilitate the development of active bacterial community, which can further harm them^14,40^. For example, we found that, at current temperatures, increasing turbidity from 4.62 to >14.2 NTUs leads to a 50% drop in the survival of *P. astreoides* recruits in the first month (Fig. 2).

The increase of sediment concentration on coral recruit survival had opposing effects in the two experiments and is expected to be due to sediment type (natural vs. anthropogenic, as intended), not recruit age. The coral recruits used in both experiments were in the same developmental stage (one-polyp), but had different ages (1 and 5 months, respectively). Due to the age difference, the effect of sediment type on coral recruit survival could not be statistically tested. However, considering that fine sediments have been repeatedly shown to be more harmful to corals than coarse sediment^40,41^, and that corals have a strong age- and size-dependent mortality, with younger individuals being more sensitive to stress^42–44^, we argue that the differences are due solely to sediment type. Since we tested the natural (theoretically, less harmful) sediment first, i.e. on the younger individuals, if anything, the differences found in the effect of sediment concentration on recruit survival between the two sediment types may be underestimated.

Increasing temperature by 4 °C led to a >20% drop in the survival of *P. astreoides* recruits, and when this was coupled with excessive anthropogenic sedimentation the negative effects were intensified. It is well known that increased temperatures cause coral mortality^26,27,45–47^. Corals evolved in a relatively stable thermal environment^7^ and thus can only persist within a relatively narrow temperature range^27,48^. Exposure to warmer temperatures increases oxidative stress in corals^36^ and causes the breakdown of the coral-algae symbiosis^29^. Higher temperatures also accelerate the metabolic rate of animals and thus lead to a faster consumption of energy reserves^35^, heightening mortality. Increasing temperature (4 °C) caused a drop in survival similar to increasing anthropogenic sedimentation (Fig. 2); when both stressors are present, coral recruits displayed even higher mortality, which indicates that these stressors have an additive effect.

The results of this study indicate that to properly understand and ultimately, regulate the deleterious effects of sedimentation, both deposited sediment concentration sedimentation and turbidity need to be monitored. Deposited sediment concentration alone is not a good indicator of impact on the coral, since whether the sedimentation effect is positive or deleterious seems to be dependent on the grain size of the sediment. Additionally, the levels of turbidity directly reflect the type of sediment that is covering the coral. When turbidity is high, it means that the sediment settled on top of the coral is fine grained and thus highly deleterious for coral recruits^14^.

From a management perspective, this study suggests that the maximum allowable turbidity in coral reefs during short-term construction and nourishment events should be less than 7 NTU. Currently, the United States Environmental Protection Agency regulates water turbidity for Florida coastal waters not to exceed 29 NTU (Nephelometric Turbidity Units) above background, during construction and nourishment operations^49^, regardless of the duration of the event. The results of this and many other studies^14,22,45^ indicate that these limits are extremely high for coral reefs. After four weeks of exposure to low turbidity (<7 NTU), 80% of *P. astreoides* recruits remained alive; exposure to turbidity levels above 29 NTU led survival to drop to 10%. The coastline of Florida is composed of different types of ecosystems, such as mangroves, seagrass beds, oyster reefs, saltmarshes, and coral reefs, with different sensitivities and resilience to fine sedimentation and turbidity. Therefore, establishing ecosystem-specific limits may prove to be a more effective way to protect natural resources which are invaluable for the local fishing and tourism industries. Recently, the dredging of the Miami Port and beach re-nourishment projects in South Florida have negatively impacted hundreds of acres of coral reefs^18,50^. Our results suggest that coral recruits were also likely negatively affected by the port dredging, undermining the recovery of the coral population^4,10^. A reduction of sedimentation in coastal areas should positively contribute to the replenishment of local coral populations after natural and human-induced global disturbances, such as severe storms and bleaching events.

Reef managers could increase coral reef resilience to ocean warming by decreasing local levels of anthropogenic sedimentation and turbidity. Corals are very sensitive to warming, but a worldwide concerted action to reduce greenhouse gas emissions is out of the sphere of action of local managers. However, coral resilience to this global stressor could be boosted by reducing the magnitude of local anthropogenic stressors that limit energy acquisition in corals, particularly when their effects are additive or synergistic. The survival of *P. astreoides* recruits under warm temperatures was maximized at the lowest anthropogenic sedimentation tested (30 mg.cm^−2^ deposited sediment, 6.55 NTU, Table 1), and is similar to the survival registered by recruits kept at current temperature (26 °C) and higher anthropogenic sedimentation (≥60 mg.cm^−2^, ≥14.2 NTU). This suggests that, if sedimentation is limited to lower levels (30 mg.cm^−2^, <7 NTU), coral resilience to ocean warming is maximized. Furthermore, the additive effect of sedimentation and warming also indicate that, when unavoidable, dredging and costal construction should be restricted to cooler times of the year. This would minimize the deleterious additive effects of sedimentation and temperature which are more harmful to coral recruits.

## Methods

### Coral Collection, and Planulae Release and Settlement

The scleractinian coral species *Porites astreoides* is a brooding species (i.e. releases fully developed competent planulae)^51^ abundant in South Florida and the Caribbean at depths of 3–20 m. Its shallow occurrence makes it vulnerable to bleaching and coastal construction^27^. Four days prior to the new moon in April 2015, eighteen adult colonies with less than 20 cm diameter were collected from two artificial patch reefs (24°44.004′ N, 80°49.577′ W and 24°43.908′ N, 80°49.634W) off Layton, in the Florida Keys (ambient seawater temperature was 26 °C, and light irradiance was ~220 μmol.m^−2^ s^−1^ at noon, measured using a Li-Cor Li250A with an Underwater Quantum Sensor LI-192). Colonies were transferred to NSU’s facilities and placed into individual 2 L plastic bowls within a recirculating 2000L outdoor tank, and supplied with filtered seawater at an average rate of ~ 1.3 L.min^−1^. Temperature and light intensity were set to mimic conditions in nature, using shade cloths and a combination of heaters and chiller. Planulae were collected using a manifold system with rubber tubing that directed water into the bowl. The water flow transported the planulae from bowl into the PVC cups fitted with plankton mesh (100 µm) at the bottom. Every morning, the cups were examined for planulae. Larval release started on the day after collection and the peak release (~5,000 planulae) occurred two days before the new moon; the number of larvae released decreased gradually until two days after the new moon. Every day, the planulae were pooled to mimic natural production in nature, transferred to 100 L tanks equipped with air pumps, kept at 26 °C and containing ca. 50–100 pre-conditioned settlement tiles (ceramic tiles with 2.5 cm diameter and 0.5 cm height kept in the reef in Broward County, FL at 8 m depth for 2 months to be colonized by bacteria and crustose coralline algae that induce larval settlement). The tiles were inspected every 8 h for planulae settlement, for a maximum of 24 h. Once larvae had settled and metamorphosed, tiles with newly settled recruits were photographed to track juvenile placement. In order to eliminate potential intraspecific effects, when corals settled in group, only one was retained for the experiment (the other(s) were eliminated). Because the quality of the larvae of brooding species can vary over time^52^, each day’s batch of newly settled recruits was randomly divided in equal numbers by all treatments, at a maximum of 50 recruits per treatment per day (i.e. all treatments have equal proportions of larvae that settled in each day). The remaining coral recruits were kept in a 2000L outdoor recirculating tank at ambient conditions (26 °C and ~220 μmol photons.m^−2^ s^−1^ at noon) and used later on in the second experiment.

To test the effects of sedimentation and temperature on the survival of coral recruits, we used a fully crossed experimental design with two temperatures, 26 °C (Spring ambient in the Florida Keys) and 30 °C (projected for 2100^25^) and four sediment concentrations, 30, 60, 90, 120 mg cm^−2^ of deposited sediment (to mimic natural to dredging levels^19^). Note that these sedimentation levels do not necessary reflect how much sediment was placed in each tank but the amount that deposited after one day (sediment was just added on the first day; one day was sufficient to cause the heaviest sediment to settle, finer sediment remained suspended due to water flow; turbidity and settled sediment were relatively constant afterwards). Each treatment was split between two 20 L tanks. The sediment was dried at 70 °C and its composition was assessed according to the Udden-Wentworth US standard classification scale^53^ prior to the experiments. Chemical analysis to the sediment were performed by Florida-Spectrum Environmental Services, Inc. Turbidity within each tank was measured in Nephelometric Turbidity Units (NTUs) using a LaMotte 2020we Turbidity Meter. The recruit tanks were equipped with two ReSun SP-800 Submersible Aquarium pumps (flow 250 L/h) to allow oxygenation and sediment resuspension, and were individually heated using temperature-regulated, programmable Aqueon Submersible Aquarium 300 W digital heaters (±0.5 °C). Temperature was monitored daily using a YSI (±0.1 °C) and was always within 0.5 °C of the desired temperature. All tanks had a 12:12 h light:dark photoperiod with Aqua Illumination Sol LED lights, with gradual increase until reaching ~230 μmol photons.m^−2^ s^−1^ between 09:00 and 14:00. Salinity was measured daily using a refractometer, and corrected with RO freshwater whenever it increased above 35. Water quality was controlled and 100% water changes were performed weekly. All individual coral recruits were monitored weekly for survival.

The first experiment was conducted using reef sediment (hereafter termed natural) and 1,503 newly settled coral recruits (ca. 188/treatment, one-polyp stage: 357–1405 µm diameter), and ran over three months, from April to July 2015. The natural sediment was collected from the upper 10–30 cm of sand near the adult colonies in the Florida Keys, and was composed by mid to large grain sizes (5% >2000 μm, 65% 500–2000 µm, and 30% 180–500 µm), and had no silt (<63 µm). After dried at 70 °C, this sediment contained a few volatile organic compounds (0.0462 mg/kg of 1,1,1,2-Tetrachloroethane and 0.0554 mg/kg of 1,1,1 – Trichloroethane) and metals (150 mg/kg of lead and 17.4 mg/kg of Zinc). With the natural sediment, turbidity levels were 0.07 to 1.01 NTU, and irradiance varied from 240 to 130 μmol photons.m^−2^ s^−1^ (at noon) from the lower to the highest sedimentation treatment, respectively (Table 3).

**Table 3:** Average turbidity and irradiance under different levels of sedimentation (deposited sediment) with natural and anthropogenic sediment.

Deposited sediment concentration (mg cm^−2^)	Natural sediment		Anthropogenic sediment	
	Turbidity (NTU)	Irradiance (μmol photons m^−2^ s^−1^)	Turbidity (NTU)	Irradiance (μmol photons m^−2^ s^−1^)
30	0.07–0.09	240	4.62–6.55	160
60	0.18–0.25	175	14.2–15.0	81
90	0.47–0.97	145	24.8–33.3	40
120	0.94–1.01	130	37.5–42.3	16

The second experiment used anthropogenic sediment and eighty 5 month old coral recruits (10/treatment, all still in the one-polyp stage: 806–2176 µm diameter), and ran over a period of one month, from September to October 2015. The sediment was collected from the upper 10–30 cm surface near the entrance of Port Everglades (hereafter termed ‘anthropogenic’ sediment) to mimic grain size composition more often found in dredging areas including ports, boat basins, and developed areas. This sediment was composed by smaller grain sizes (35% 180–500 µm, 45% 63–180 µm, and 20% <63 µm) and contained volatile organic compounds (0.0100 mg/kg of 1,1,1,2-Tetrachloroethane and 0.0119 mg/kg 1,1,1-Trichloroethan), metals (9.12 mg/kg of Lead and 68.9 mg/kg of Zinc) and several polycyclic aromatic hydrocarbons (PAHs) (0.00471 mg/kg Acenaphthene, 0.0115 mg/kg Anthracene, 0.0779 mg/kg Benzo (a) anthracene, 0.0669 mg/kg Benzo (a) pyrene, 0.124 mg/kg Benzo (b) fluoranthene, 0.0793 mg/kg Benzo (g,h,i) perylene, 0.0551 mg/kg Benzo (k) fluoranthene, 0.0826 mg/kg Chrysene, 0.0237 mg/kg Diberit (a,h) anthracene, 0.146 m/kg Flouranthene, 0.0663 mg/kg Indenol (1,2,3,cd) pyrene, 0.00478 mg/kg Naphthalene, 0.0224 mg/kg Phenanthrene, and 0.134 mg/kg Pyrene). With the anthropogenic sediment, turbidity levels varied from 4.6 to 42.3 NTU, and irradiance varied from 160 to 16 μmol photons.m^−2^ s^−1^  (at noon) from the lower to the highest sedimentation, respectively (Table 3). Since this experiment was only planned after the results of the first experiment were obtained, it was impossible to use recruits of the same age (although they were all in the dame developmental stage: one-polyp) and equal number of replicates, thus the experiments were analyzed separately and results were not statistically compared.

### Data Analysis

For each experiment, we used survival analysis to test the synergistic effect of temperature and sedimentation on the survival of *P. astreoides* recruits. Specifically, we used the Kaplan-Meier estimator to describe the survival curves, and a Cox model to assess the effects of temperature and sedimentation coral recruit survival. To determine if the survival of coral recruits reared in warmer conditions could be ameliorated under lower sedimentation, we did pairwise comparisons of the survival curves using Mantel-Haenszel (log-rank) tests. Since the second experiment was only planned after the results of the first experiment were obtained, the experiments were analyzed separately and results were not directly compared, i.e. in each experiment we could only test the effects of different degrees of sedimentation on survival, but the data of the two experiments was not combined to test the effect of sedimentation type (natural vs. anthropogenic) on recruit survival. The survival analysis was conducted in Software R, version 3.2.1 using package “survival”^54^.

### Data Availability

The datasets generated during the current study are available in  dryad.org repository: 10.5061/dryad.4t85s.
